# The Book World of Medicine and Science

**Published:** 1911-08-19

**Authors:** 


### The Book World of Medicine and Science.

Surgical Instruments and Appliances. By Harold Burrows, F.R.C.S. Third edition. (London: The Scientific Press, Ltd. Price 1s. 6d. net.)

The object of this little book is to put briefly before the practitioner the instruments he will require in performing any operation which may be undertaken in general practice, and in this respect it is most excellent, both for the way it is written and the profuse number of illustrations. By far the best section is that on the preparation for an operation on a patient in a private house. The reading of this by any medical man must needs prove most useful, for both the preparing of the patient and of the room are most admirably dealt with. The work reflects credit on the author and publishers alike.

Sight-testing Made Easy. By W. Wright Hardwicke, M.D. St. And., M.R.C.P., L.R.C.S. Ed. (London: J. and A. Churchill, 1910. 2s. 6d. net.)

Originally intended to be a guide to the practitioner desirous of testing for refractive defects by the subjective method, the author has seen fit in this, the second, edition to add a chapter on retinoscopy. There is no denying that in the case of patients who are compelled by the necessity of earning their living to use their accommodation frequently it is almost impossible to insist on the use of a mydriatic unless the need for so doing is urgent. In this class of patients subjective eye-testing is of decided advantage. Unfortunately, however, the glasses the patient prefers are not always those which he ought by right to be wearing, and the practitioner can only get an approximate idea of the right glasses by this method. Retinoscopy without a mydriatic is by no means easy unless the surgeon has had a good deal of practice in refraction work. On the whole, therefore, it would seem best for the general practitioner—and it is for him the book has been written—to insist on the use of a mydriatic by the patient prior to retinoscopy in all cases in which it is convenient, and always in the case of children. We think, therefore, that Dr. Hardwicke's new chapter is entirely in place, and that the value of the book has been much enhanced by its addition. The little work is in every way reliable, straightforward, and clear, and we heartily recommend it as a preliminary to a perusal of more advanced works on refraction.

The Death-dealing Insects and Their Story. By C. Conyers Morrell. (Manchester: H. A. W. Offices. 1910. Pp. 79. With map and illustrations. Price 1s. net.)

This brief account of the life-history and habits of the various parasites of man and animals which are responsible respectively for the spread of malaria, elephantiasis, sleeping sickness, plague, relapsing fever, as well as of a number of diseases occurring among cattle and sheep in tropical regions, can be recommended to the notice of the general public and more particularly to that portion of it whose duties necessitate their living in countries where the presence of such parasites is a menace to the life of man and beast. It is only by educating the public as to the factors which are responsible for these diseases that an intelligent co-operation with the efforts of medical officers in the tropics towards prophylactic measures can be secured. So long as a panic exists the layman can be relied on to take precautions, but when the danger is passed or checked the efforts of yesterday are relaxed and the enemy once again gets his chance of success. Books, such as the one now under notice, which give accurate information and advice at a price within the reach of all, and, moreover, which explain the reason why precautions are necessary, are of the utmost value to the success of what Dr. Morrell calls "the great campaign"—namely, that against mosquitoes. It is only by the publication and dissemination of such works that the mischievous activities of various bodies and associations whose object would seem to be the vilification of the devoted labours of medical scientists, can be combated, and it is for this reason that we welcome the appearance of this book. Written in simple language, with all technical terms explained *currente calamo*, and illustrated with simple and clear diagrams, it should prove interesting even to readers whose knowledge of things medical is of the slightest.

